# A Review of the Impact of Pharmacogenetics and Metabolomics on the Efficacy of Metformin in Type 2 Diabetes

**DOI:** 10.7150/ijms.77206

**Published:** 2023-01-01

**Authors:** Zoheir A. Damanhouri, Huda M. Alkreathy, Fawaz A. Alharbi, Haneen Abualhamail, Muhammad S. Ahmad

**Affiliations:** 1 Pharmacology Department, Faculty of Medicine, King Abdulaziz University, Jeddah, Saudi Arabia.; 2 Department of Veterinary Medicine, University of Cambridge, Cambridge, United Kingdom.

**Keywords:** Type 2 diabetes, Metformin, Pharmacogenetics, Metabolomics, Metformin Transporters.

## Abstract

Metformin is the most often prescribed drug for people with type 2 diabetes (T2D). More than 120 million patients with T2D use metformin worldwide. However, monotherapy fails to achieve glycemic control in a third of the treated patients. Genetics contribute to some of the inter-individual variations in glycemic response to metformin. Numerous pharmacogenetic studies have demonstrated that variations in genes related to pharmacokinetics and pharmacodynamics of metformin's encoding transporters are mainly associated with metformin response.

The goal of this review is to evaluate the current state of metformin pharmacogenetics and metabolomics research, discuss the clinical and scientific issues that need to be resolved in order to increase our knowledge of patient response variability to metformin, and how to improve patient outcomes. Metformin's hydrophilic nature and absorption as well as its action mechanism and effectiveness on T2D initiation are discussed. The impacts of variations associated with various genes are analysed to identify and evaluate the effect of genetic polymorphisms on the therapeutic activity of metformin. The metabolic pattern of T2D and metformin is also indicated. This is to emphasise that studies of pharmacogenetics and metabolomics could expand our knowledge of metformin response in T2D.

## Introduction

*Diabetes mellitus* (DM) commonly known as diabetes is on the rise worldwide. According to the International Diabetes Federation's most recent estimate, there were roughly 537 million diabetics worldwide in 2021, and that number is projected to increase to 643 million by the year 2030 and 783 million by the year 2045 1, 2. This equates to 1 in 10 adults aged between 20 and 79 years in the world suffering from diabetes. In addition, 541 million individuals were anticipated to be at risk of becoming diabetic in 2021 because of their impaired glucose tolerance. This results in extreme healthcare costs in addition to the enormous personal and social costs. Indeed, total diabetes healthcare costs were estimated to be nearly one trillion USD and will exceed this amount by 2030 2. According to the World Health Organization (WHO), diabetes was the ninth leading cause of death worldwide in 2019 3. Moreover, recent studies have revealed that patients with diabetes had a 2.3-fold higher risk of death and a 3.6-fold higher likelihood of being hospitalised with COVID-19 than those without diabetes 4-6. All this adds up to demonstrate the magnitude of the issue we face in the form of a global diabetes epidemic.

The prevalence of diabetes is growing at a fast rate in Saudi Arabia. In total, one-fourth of the adult population is affected by diabetes, and the number is anticipated to double by 2030. The most concerning trend is the rise in diabetes prevalence in recent years, with a nearly ten-fold increase in Saudi Arabia during the previous thirty years 7. Recently, the findings of a multi-center, retrospective, cross-sectional study conducted nationwide found that a significant number of COVID-19 patients (7.6 to 68.3%) had diabetes 8. Diabetes accounts for 24 percent of the nation's total healthcare spending, making it the highest per capita spending state in the Middle East and North Africa 2.

Ninety to ninety five percent of all diabetes cases are caused by type 2 diabetes (T2D). Despite at least eleven other classes of drugs being used for the same condition, metformin is still the most recommended treatment (80%-85%) for people with T2D. Most medical associations, including the International Diabetes Foundation and the American Diabetes Association, recommend metformin as the primary oral glucose-lowering medication in addition to lifestyle control to treat T2D 1, 9, 10. Previous studies have shown that metformin was prescribed as first-line monotherapy to over 120 million T2D patients worldwide 11, to 73 percent of T2D patients in the UK between 2000 and 2017, and to 89 percent of T2D patients in 2017 12. Metformin is the drug of choice for T2D patients who are overweight because it reduces weight gain and the risk of microvascular problems 13. Furthermore, metformin use was associated with a continued decrease in microvascular risk, as well as a decrease in the risk of myocardial infarction in prediabetic patients 14. It is also a popular treatment for pre-diabetes, gestational diabetes, and polycystic ovary syndrome 15. Some studies have shown that in patients with chronic kidney disease (stage 3), metformin is contraindicated and users with kidney damage are more likely to develop lactic acidosis 16. The risk of most causes of death and incidence of terminal renal disease was, however, shown to be reduced by metformin in individuals with chronic kidney disease and renal fibrosis, according to recent research 17, 18. The disparity between the various studies is due to the complex renal effects of metformin, which depend on the kind of disease and also the type and onset of the injury.

The pharmacological response to metformin varies significantly across individuals. Earlier studies showed that metformin did not achieve the optimal glycemic regulation in 35% of patients, necessitating dose escalation or the use of a combined hypoglycemic medication 19, 20. In children and adolescents with newly diagnosed T2D, the failure rate was as high as 50% 21. Other studies indicated that African Americans had better glycemic regulation with metformin than European Americans 22.

The study of pharmacogenetics plays a constructive role in identifying the crucial genetic factors that significantly influence typical drug responses in the body. The high polymorphisms rate associated with certain genes generally accounts for subsequent drug responses in certain individuals. Certain variations can either enhance or reduce the overall exposure and activity of a drug in the body; therefore, study of these factors should be necessarily taken into account to achieve the required responses in patients 23. Moreover, studies examining links between metabolites and T2D risk and symptoms may also shed light on the disease's pathophysiological processes and lead to the discovery of new, reliable biomarkers that will help T2D be detected and treated more effectively 24, 25.

The primary goals of this review are to advance mechanistic knowledge of metformin's pharmacokinetics and pharmacodynamics in T2D and to examine the effects of its pharmacogenetics and metabolomics.

## Pharmacological aspects of metformin

### Metformin transporters involved in its pharmacokinetics

Metformin is excreted in its whole form in urine. The drug's hydrophilic nature prevents it from diffusing across cell membranes, so it must rely on organic cation transporters for active transport into and out of enterocytes, hepatocytes, and renal epithelial cells 26.

After oral intake, metformin is absorbed by enterocytes from the gut endothelium through Solute Carrier Family 29 Member 4 (*SLC29A4*), plasma monoamine transporter (PMAT), organic cation transporter 1 (OCT1; *SLC22A1*), OCT3 (*SLC22A3*) and OCTN1 (*SLC22A4*) located on the inner surface of the gut epithelium. Additionally, SERT (*SLC6A4*) and THTR-2 (*SLC19A4*) may contribute to the intestinal absorption of metformin, whereas OCT1 transports it into the bloodstream. OCT1 and OCT3 both take metformin from the bloodstream and store it in hepatocytes 27, 28. Polyspecificity is one of the OCT1's most distinguishing characteristics 29. Currently, it has been determined that 150 cationic organic molecules of various chemical compositions, including well used medications like metformin, are substrates for the OCT1 30-32.

Metformin is transported into kidney epithelial cells by OCT1 (*SLC22A1*) and OCT2 (*SLC22A2*). Additionally, the disposal of metformin from proximal tubule cells into the urine is carried out by the multidrug and toxin extrusion 1 protein (MATE1) encoded by the gene (SLC47A1) and MATE2 encoded by the gene (SLC47A2), while MATE1 (SLC47A1) is also in charge of the disposal of metformin from hepatocytes into the bile 27, 33. Furthermore, OCT3 is expressed in many organs such as the lungs, kidney, placenta, prostate and salivary glands, where it may play a role in metformin's transportation 34, 35. ATP-binding cassette (ABC) transporters P-glycoprotein (P-GP) and the breast cancer resistance protein (BCRP) transporters have been shown to be mainly responsible for foetal-to-maternal efflux of metformin in gestational diabetes subjects 36. A summary of various organ transporters involved in the pharmacokinetics of metformin is presented in Table 1. When metformin is given orally, half of it is lost in the systemic circulation and has a plasma half-life of 4 to 8 hours in individuals with normal renal function 37, 38.

### Metformin pharmacodynamics

Although metformin has been the most successful treatment for T2D for more than 60 years, its specific action is still uncertain. Metformin is thought to regulate blood sugar levels through pleiotropic processes involving multiple pathways 38, 39. The drug works by decreasing glucose production from the liver by inhibiting hepatic gluconeogenesis. Metformin has been shown to act through the activation of AMP-activated protein kinase (AMPK), an enzyme involved in cellular regulation of energy homeostasis, as well as lipid and glucose metabolism, however, AMPK independent mechanisms have also gained interest recently 39. Earlier studies had shown that metformin inhibited mitochondrial Complex I 40, 41. This was believed, at least in part to contribute to prevent hepatic glucose production and increase glucose utilization in peripheral tissues. A recent study confirmed that metformin inhibits mitochondrial Complex 1; prevent mitochondrial ATP production, thereby, increase cytoplasmic AMP:ATP and ADP:ATP ratios and thus activate AMPK dependent-pathways 42. Higher AMP:ATP ratio or compromised cellular energy balance has been shown to inhibit fructose-1,6-bisphosphatase and thereby causing acute inhibition of gluconeogenesis 43. Activated AMPK regulates energy homeostasis by phosphorylation of acetyl-CoA carboxylase 1 and 2, thereby inhibiting fat synthesis, accelerating fat oxidation and depletion of fat stores, ultimately improving insulin sensitivity 44. Gluconeogenesis is an energy intensive process that requires 6 molecules of ATP per molecule of glucose produced. Therefore, metformin inhibition of ATP production explains its inhibition of gluconeogenesis. Moreover, changes in the NAD+:NADH ratio resulting from respiratory chain inhibition by metformin are also believed to contribute towards inhibition of gluconeogenesis. Mitochondrial glycerophosphate dehydrogenase, a redox shuttle enzyme, has also been proposed as a mitochondrial target of metformin 45, however, further studies are warranted to establish its role in inhibition of gluconeogenesis. Multiple mechanisms of metformin action especially its effects on energy metabolism are reviewed in 39.

Metformin also affects the function of AMPK through the serine/threonine kinases Ataxia-telangiectasia (*ATM*) and serine threonine kinase 11 (*STK11*). In liver cells, activated AMPK reduces the chances of fat diseases by directly repressing lipogenesis and cholesterol biosynthesis, which is mainly achieved by enhancing the fatty acid beta-oxidation and mitochondrial biogenesis in the body. Metformin's pleiotropic effects can help to reduce diabetes complications, especially cardiovascular diseases 26. In the skeletal muscle cells, metformin activates AMPK to promote glucose uptake by increasing *SLC2A4* encoded glucose transporter type 4 (GLUT-4) translocation 46, 47.

In gut, metformin increases glucose uptake, glycolytic lactate production, glucagon-like peptide-1 (GLP-1) secretion and bile acid pool in addition to microbiome alterations thus having pleiotropic effects on blood glucose homeostasis 48, 39. Recent studies have shown that metformin treatment leads to changes in gut microbiota and relative abundance of microbial metabolites 49,50. Cumulative evidence from those reports confirms that hypoglycemic effects of metformin were associated with AMPK activation and microbial metabolites involved in energy metabolism, gluconeogenesis and branched-chain amino acid metabolism. To fully understand how metformin works in the gut, however, further research is required.

### Metformin pharmacogenetics

Previous studies have greatly focused on drawing associations among various genomic factors and their impacts on drug responses. In the case of metformin, OCT1 is highly polymorphic and is preferentially encoded by the gene *SLC22A1*. This transporter protein is greatly expressed in the liver and few other body cells. Interindividual differences in metformin responses among T2D patients can be attributed to the high polymorphisms associated with OCT1. A loss of functional variant associated with OCT1 has been linked to lower metformin response in some patients 24, 51. Four functional variations (rs12208357/R61C, rs72552763/M420del, rs34059508/G465R, and/or rs34130495/G401S) were connected to a decreased metformin's ability to lower blood sugar in healthy human volunteers following an oral glucose tolerance test (OGTT), according to research by Shu and colleagues 52. Reduced functional variants of OCT1 (rs72552763/M420del, rs12208357/R61C, rs34059508/G465R, and rs34130495/G401S) were also associated with trough concentrations of metformin and improvement in HbA1c after six months of metformin treatment in a cohort of 151 T2D patients in a prospective multicentre South Danish Diabetes Study 53. In a study of 1,915 completely metformin-tolerant and 251 intolerant T2D patients, allelic variants of OCT1 rs12208357/R61C, rs72552763/M420del, rs34130495/G401S, rs55918055/C88R, and rs34059508/G465R were found to be related to metformin intolerance due to increased metformin accumulation in enterocytes 54. Later studies reported three variants of OCT1 (rs622342, rs628031, and rs594709) that showed overall reduction in the efficacy of metformin among various populations 55, 56. Metformin hepatic accumulation was also found to be decreased in humans carrier of rs12208357/R61C and rs72552763/M420del *SLC22A1* forms, although the blood levels of metformin were unaltered 24. OCT1 gene polymorphisms, however, may not have a significant impact on the clinical effectiveness of metformin, according to a prior study by Shikata et al. 57. Similar to this, the GoDARTS research revealed no association between the two most prevalent OCT1 variations in terms of allelic frequency in persons of European descent—rs12208357/R61C and rs72552763/M420del—with glycaemic response to metformin in a sample of 1,531 patients with T2D 58. Therefore, it is imperative to say that some OCT1 variants have effects on metformin response but the small number of patients and the co-medication of other anti-hyperglycaemic drugs in some study groups represent a drawback and explain the discrepancies between the various studies.

Several studies have found no evidence of a connection between OCT2 variants (encoded by the gene *SLC22A2*) and metformin response 24, 51, 59, 60. However, it has been demonstrated that the particular C allele of the variant rs8192675 in the gene *SLC2A2*, which codes for the glucose transporter (GLUT2), is crucial for controlling the metformin action 61. Reduced metformin uptake and altered substrate selectivity were present in OCT3 variants T44M (c.131C>T), V423F (c.1267G>T), and T400I (c.1199C>T), particularly in the latter two 20.

Inconsistent findings have been reported for metformin response by variants of *SLC47A1* gene encoding MATE1 53, 60, 62, 63. According to studies, homozygous carriers of the *SLC47A1* rs2289669 A-allele have a much greater decrease in HbA1c following a six-month therapy with metformin than do carriers of the more prevalent G-allele. Therefore, approximately 20% of T2D patients showed a higher two-fold (0.55%) reduction in HbA1c levels than the rest of the patients 63. However, meta-analysis of Metformin Genetics Consortium studies involving nearly 8,000 T2D patients found no substantial association between glycaemic response to metformin and MATE1 transporter genes besides other genes encoding OCT1, OCT2, MATE2-K, and OCTN1 transporters 60. A recent pharmacogenetics study found no association of metformin transporters OCT1, OCT2, OCT3 and P-GP with therapeutic inefficacy among Mexican T2D patients 36.

The transcription factor 7-like 2 (*TCF7L2*) gene has been demonstrated to be closely related with T2D through poor glucose control and insulin production, in addition to genes that encode transporter proteins and influence responses in patients with T2D 64. Physiological investigations have also indicated that rs290487 variant played a role in insulin resistance, implying that Wnt signalling is involved in insulin resistance 65. Furthermore, *TCF7L2* variants were also linked to a faster decline in pancreatic ß-cell function, higher glycemic levels, and lower insulin production, all of which are linked to familial history of T2D 66. Other studies have shown that the *TCF7L2* rs 7903146T genotype is associated with both T2D and obesity, and that carriers may have changes in their plasma metabolic profiles due to phospholipids, suggesting that phospholipids may cause metabolic abnormalities prior to the onset of glucose intolerance 67, 68. These effects were recently brought to light by research demonstrating that the *TCF7L2* gene controls a number of processes, including adipogenesis and disorders like T2D, as a downstream effector of the Wnt/ß-catenin signaling system 69.

From these various studies there seems to be lack of consistency in molecular pathways, and the need for more functional studies on metformin pharmacodynamics. Known metformin pharmacokinetic and pharmacodynamic gene variants significantly linked to metformin clinical response are presented in Table 2.

### Metformin and metabolomics studies in T2D

The systematic identification and measurement of all metabolic products in the human body is known as metabolomics. This field has the potential to give researchers with new diagnostic biomarkers for disease states, as well as assessing therapy response to drugs on an individual basis. A systematic review and meta-analysis of cross-sectional and prospective human metabolomics studies in prediabetes and T2D patients showed higher levels of carbohydrates (glucose and fructose), lipids (phospholipids, sphingomyelins, and triglycerides), and amino acids (branched-chain and aromatic amino acids)) in T2D patients compared to control subjects 79. Meta-analysis of prospective studies provided evidence that people with higher levels of isoleucine, leucine, valine, tyrosine and phenylalanine had higher risk of developing T2D while increased glycine and glutamine levels were found to be associated with lower risk of developing T2D 79. The authors reported that multiple (more than 10) studies reported positive correlation of sugar metabolites dihexose, glucose, mannose, fructose, and arabinose with T2D. Another study reported that 1,5-anhydroglucitol plasma levels were about 37.8% lower in T2D patients while mannose, glucose, deoxyhexose, and di-hexose levels were significantly higher in diabetic patients than in controls 80. One of the endogenous substrates for OCT2 has been discovered as tryptophan, and it has the potential to serve as a biomarker candidate for the variability of OCT2's transport activity 81. Along with a number of organic substances, such as purines and metabolites of the urea cycle, such as ornithine, arginine, and citrulline, several organic acids, such as maleic acid, acetic acid, and dimethyl ester, have also been linked to T2D. 79, 82. Studies have also investigated the relationship between T2D and branched chain amino acids leucine, isoleucine, and valine, and discovered that high levels of these amino acids are linked to insulin resistance in overweight children 83. Other studies have reported that an increase in the levels of branched-chain amino acids is a specific and reliable indicator of future insulin resistance in T2D patients 79, 84. Moreover, Yoon, (2016) have found that patients with T2D have greater plasma concentrations of aromatic and branched-chain amino acids, as well as a higher glutamate to glutamine ratio, than healthy people 82. Amino acids (including hydroxy acids and hydroxybutyrate) have been associated to increased insulin resistance and decreased glucose tolerance in diabetic individuals, whereas 3,hydroxybutyrate and hydroxybutyrate have been connected to a greater risk of prediabetes 85. Metformin treatment of obese T2D patients for 6 months resulted in 30 dysregulated (21 were up-regulated and 9 were down-regulated) metabolites mainly related to amino acids metabolism to change to obese control levels. Metformin treatment may have adjusted the dysregulated pathways in T2D or those dysregulated pathways may be common for both T2D and metformin. However, 71 dysregulated (30 up-regulated and 41 down-regulated) metabolites remained unchanged after metformin treatment 86. The study was underpowered due to smaller sample size. Similar large-scale targeted and untargeted metabolomics studies using a multi-platform approach could unravel novel molecular targets of metformin in T2D patients. Numerous glutamine-containing dipeptides, such as glutathionyl-L-cysteine and creatine, were found to be dramatically elevated in T2D. Those included glutaminyl-glutamine, glutamyl-glutamine, glutaminyl-glutamic acid, tyrosyl-glutamine, and glutamyl-tyrosine and then returned to obese comparable levels after metformin treatment 86. Given previously unestablished effect of metformin on glutamine metabolism, further studies are required to validate this finding. The 10 dysregulated metabolites only observed after metformin treatment may be involved in pharmacodynamics of metformin and shows the power of metabolomics studies in understanding drug action. The glycolytic pathway intermediate 3,phosphoglycerate (3PGA), which is a source of the metabolite serine, was downregulated in T2D, suggesting a potential change in the disease's metabolic route.

Adam and colleagues in a well-defined German Kooperative Gesundheitsforschung in der Region Augsburg (KORA) cohort looked at the metabolite profile of T2D patients undergoing metformin treatment 87. They discovered citrulline and an unknown metabolite associated with metformin treatment in T2D patients using a non-targeted mass spectrometry approach. This finding was repeated in mice and confirmed in a follow-up cohort. The same group had discovered that T2D patients taking metformin had slightly lower ornithine levels 88. The authors speculated that metformin/AMPK/eNOS/NO could mediate increased citrulline excretion in urine, resulting in lower citrulline levels in serum. Recent research has demonstrated that the metformin-treated diabetic individuals in both the lean and obese groups had lower levels of taurine, citrulline, and 5-hydroxymethyluracil (HMU), although levels of salicylic acid, L-proline, and L-alanine were increased 86. According to the same study, guanido-acetic acid and L-arginine were among the down-regulated metabolites in the metformin-treated patients with T2D in the lean and obese groups. Contrarily, the metabolites of the urea cycle, such as homoarginine and citrulline, the methylene homologue of arginine, are down-regulated in the obese T2D group receiving metformin. Some metabolic patterns as an effect of prediabetes, diabetes and also as metformin therapy in T2D are shown in Table 3.

Taken together, above studies have shown the value of using metabolomics methods to identify potential targets and molecular mechanism in metformin pharmacodynamics in T2D patients.

## Conclusion

This article describes the importance of determining the pharmacogenetics and metabolomics for interindividual differences in metformin response. Exploration of metformin's clinical omics will not only lead to better prescribing, but it will also help to explain the pleiotropic mechanisms by which metformin works. Various pathways implicated in the effects of T2D drugs are possibly multifactorial. However, studies have demonstrated the value of metabolomics in exploring the pharmacodynamics of metformin in patients with T2D. Genes with metformin response polymorphisms suggest that genetic factors may play a significant role in the therapeutic response to T2D treatment. Moreover, this review provides ample evidence about the association between various transporter genes polymorphism and its significant impact on altered metformin response in T2D patients. There is a need for more research in sizable cohorts of previously examined patients from various ethnic/genetic and cultural backgrounds. Future development of specialized tools for improved therapy will be made possible by a greater knowledge of metformin metabolomics, T2D, pharmacogenetics, and frequent and unusual gene mutations in T2D patients.

## Figures and Tables

**Table 1:** A Summary of Various Transport Proteins with Tissue-Specific Expression Pattern Involved in Pharmacokinetics of Metformin.

Organ/Cells	Transporters (Gene)	Reference
Intestinal Cells	OCT1 (*SLC22A1*) OCT3 (*SLC22A3*) PMAT (*SLC29A4*) OCTN1 (*SLC47A4*) THTR-2 (*SLC19A4*) SERT (*SLC6A4*)	(15, 27)
Liver Cells	OCT1 (*SLC22A1*) OCT3 (*SLC22A3*) MATE1 (*SLC47A1*)	(15, 27, 28)
Kidney Cells	OCT1 (*SLC22A1*) OCT2 (*SLC22A2*) MATE1 (*SLC47A1*) MATE2 (*SLC47A2*)	(15 , 27)
Skeletal Cells	OCT3 (*SLC22A3*) OCTN1 (*SLC47A4*)	(15, 27)
Placenta /Lung /Prostate/Salivary Glands	OCT3 (*SLC22A3*) P-GP (*ABCB1*) BCRP (*ABCG2*)	(28, 34, 35, 36)

**Table 2:** Known metformin pharmacokinetic and pharmacodynamic gene variants (SNPs) significantly linked to metformin clinical response.

Gene	dbSNP ID	Cohort	Reference
SLC22A1 (OCT1)	rs1867351 rs4709400 rs628031 rs2297374	Han Chinese 153 T2D 124 Control	(55)
		256 healthy brazilian	(70)
	rs622342	125 Chinese	(71)
		63 Lebanese patients	(72)
		256 Brazilian healthy	(70)
	rs12208357 rs72552763 rs34059508	256 healthy brazilian	(70)
	rs34130495 rs461473 rs34104736	371 Danish T2D patients	(53)
	rs594709	267 T2D Chinese patients and182 healthy subjects	(56)
SLC22A2(OCT2)	rs315978 rs662301	2,994 Participants in Diabetes Prevention Program	(62)
	rs316019	125 Chinese participants	(71)
		256 Brazilian adults	(70)
		91 Korean subjects	(73)
	rs201919874	400 Pakistani patients	(74)
SLC22A3 (OCT3)	rs3127602	258 White Europeans	(75)
	rs520685 rs520829	25 Korean subjects	(76)
SLC29A4 (PMAT)	rs10234709	258 White Europeans	(75)
	rs2685753	91 Korean subjects	(73)
	rs3889348 rs4720572 rs4299914 rs6971788	91 Korean subjects	(73)
SLC47A1**(MATE1)**	rs2289669	267 T2D Chinese patients and 182 healthy subjects	(56)
		125 Chinese subjects	(71)
		256 Brazilian subjects	(70)
		91 Korean subjects	(73)
	rs8065082 rs2453583	2,994 DPP	(62)
	rs2120274	258 White Europeans	(75)
	rs2252281	125 Chinese subjects	(71)
		256 Brazilian subjects	(70)
SLC47A2**(MATE2)**	rs4621031	258 White Europeans	(75)
	rs34399035	371 Danish T2D Patients	(53)
	rs12943590	256 Brazilian subjects	(70)
		91 Korean subjects	(73)
	rs34834489	91 Korean subjects	(73)
	Rs138244461	400 Pakistani patients	(74)
STK11	rs2301759	258 White Europeans	(75)
	rs2075604	Chinese 94 T2DMpatients	(71)
ATM	rs1800058	258 White Europeans	(75)
	rs11212617	460 Russian T2DM patients	(77)
TCF7L2	rs7903146	525 Taiwanese subjects	(65)
		1023 Brazillian subjects	(68)
ABCB1(P-GP)	rs1128503 rs2032582	103 Mexican DMT2 patients	(78)

**Table 3:** Some metabolic patterns as an effect of prediabetes, diabetes and metformin therapy in T2D (**↑**: up-regulated, **↓**:down-regulated).

Prediabetes (25)	Diabetes (25)	Metformin in T2D (86)
Branch-chain amino acids **↑**	Branch-chain amino acids	L-arginine **↓**
Aromatic amino acids	Aromatic amino acids	guanidoacetic acid **↓**
Glutamine/glutamate ratio	Glutamine/glutamate ratio	L-proline **↑**
B-Hydroxybutyrate **↑**	Mannose **↑**	taurine **↓**
	glucose **↑**	L-alanine **↑**
	sugar metabolites **↑**	citruline **↓**
	organic acid **↑**	5-hydroxymethyluracil **↓**
	1,5,Anhydroglucitol **↓**	Salicylic acid **↑**
